# Insecticide-treated net use before and after mass distribution in a fishing community along Lake Victoria, Kenya: successes and unavoidable pitfalls

**DOI:** 10.1186/1475-2875-13-466

**Published:** 2014-11-28

**Authors:** Peter S Larson, Noboru Minakawa, Gabriel O Dida, Sammy M Njenga, Edward L Ionides, Mark L Wilson

**Affiliations:** Department of Epidemiology, School of Public Health, University of Michigan, 1415 Washington Heights, Ann Arbor, MI 48109-2029 USA; Department of Vector Ecology and Environment, Institute of Tropical Medicine (NEKKEN) and the Global COE Program, Nagasaki University, 1-12-4 Sakamoto, Nagasaki, Nagasaki, 852-8523 Japan; Department of Biomedical Science and Technology, School of Public Health, Maseno University, Maseno, Kenya; Eastern and Southern Africa Centre of International Parasite Control, Nairobi, Kenya; Department of Statistics, University of Michigan, 429 West Hall, Ann Arbor, MI 48109-1107 USA

## Abstract

**Background:**

Insecticide-treated nets (ITNs) have proven instrumental in the successful reduction of malaria incidence in holoendemic regions during the past decade. As distribution of ITNs throughout sub-Saharan Africa (SSA) is being scaled up, maintaining maximal levels of coverage will be necessary to sustain current gains. The effectiveness of mass distribution of ITNs, requires careful analysis of successes and failures if impacts are to be sustained over the long term.

**Methods:**

Mass distribution of ITNs to a rural Kenyan community along Lake Victoria was performed in early 2011. Surveyors collected data on ITN use both before and one year following this distribution. At both times, household representatives were asked to provide a complete accounting of ITNs within the dwelling, the location of each net, and the ages and genders of each person who slept under that net the previous night. Other data on household material possessions, education levels and occupations were recorded. Information on malaria preventative factors such as ceiling nets and indoor residual spraying was noted. Basic information on malaria knowledge and health-seeking behaviours was also collected. Patterns of ITN use before and one year following net distribution were compared using spatial and multi-variable statistical methods. Associations of ITN use with various individual, household, demographic and malaria related factors were tested using logistic regression.

**Results:**

After infancy (<1 year), ITN use sharply declined until the late teenage years then began to rise again, plateauing at 30 years of age. Males were less likely to use ITNs than females. Prior to distribution, socio-economic factors such as parental education and occupation were associated with ITN use. Following distribution, ITN use was similar across social groups. Household factors such as availability of nets and sleeping arrangements still reduced consistent net use, however.

**Conclusions:**

Comprehensive, direct-to-household, mass distribution of ITNs was effective in rapidly scaling up coverage, with use being maintained at a high level at least one year following the intervention. Free distribution of ITNs through direct-to-household distribution method can eliminate important constraints in determining consistent ITN use, thus enhancing the sustainability of effective intervention campaigns.

**Electronic supplementary material:**

The online version of this article (doi:10.1186/1475-2875-13-466) contains supplementary material, which is available to authorized users.

## Background

Insecticide-treated bed nets (ITNs) have proven instrumental in the fight against malaria in sub-Saharan Africa (SSA) [1, 2]. The World Health Organization (WHO) has recommended that all health ministries and donor agencies scale up the distribution of ITNs, specifically to target populations of small children and pregnant women [3]. ITNs have been shown to be associated with an average 20% reduction in overall parasite prevalence across a number of geographic contexts [2] accompanied by precipitous drops in certain *Anopheles* vector species in Kenya [4].

However, despite massive scale-up of ITN distribution all over SSA, shortfalls and inequities exist [5], which might compromise long-term elimination or control programmes. Possession of ITNs has been shown to be associated with factors such as proximity to distribution sites, cost, socioeconomic status, and the method of distribution [6–10]. ITN distribution programmes can rapidly increase ownership and bolster household use. In Sierra Leone, for example, a mass distribution campaign increased household use 137% within six months [11]. Possession, however, does not necessarily imply proper use. Whether ITNs are effectively used to prevent malaria depends on a complex set of factors. In one Ghana study, people used ITNs to reduce the nuisance of mosquitoes, and not to prevent malaria [12]. In the western Kenyan highlands, another study showed that seasonal patterns of precipitation and vector density, along with education, were associated with ITN use [13]. Sleeping arrangements, such as sleeping on the floor (as opposed to a bed), and availability of areas amenable to hanging nets also have been shown to be associated with ITN use [14]. Comprehensive knowledge of malaria risk factors, and education about the purpose of ITNs has increased use among pregnant women in Nigeria [15].

Challenges to maintaining consistent coverage and compliance following ITN distribution scale-ups have been presented in various other investigations. One study in Burkina Faso noted declines in motivation less than one year following widespread distribution, citing problems of convenience and the perception that malaria is multi-factorial [16]. Leakage of freely distributed nets (loss or disappearance of ITNs following distribution) was seen in a Senegal study, with nearly 10% of nets provided to the community being absent from target households six months later [17]. Leakage has also been shown to compromise the cost-effectiveness of distribution campaigns [18]. To confront challenges that ensure widespread compliance, research has suggested that providing even minimal education can effectively increase household use compared with other methods [19, 20].

To further evaluate the complex interaction of factors that impact effective ITN scale-up, the current project investigated patterns of ITN use before and one year following a mass distribution campaign. This research explored determinants of use at the individual, household and community level in order to uncover factors that may compromise future, sustained efforts to expand ITN ownership and use.

## Methods

**Figure 1:**
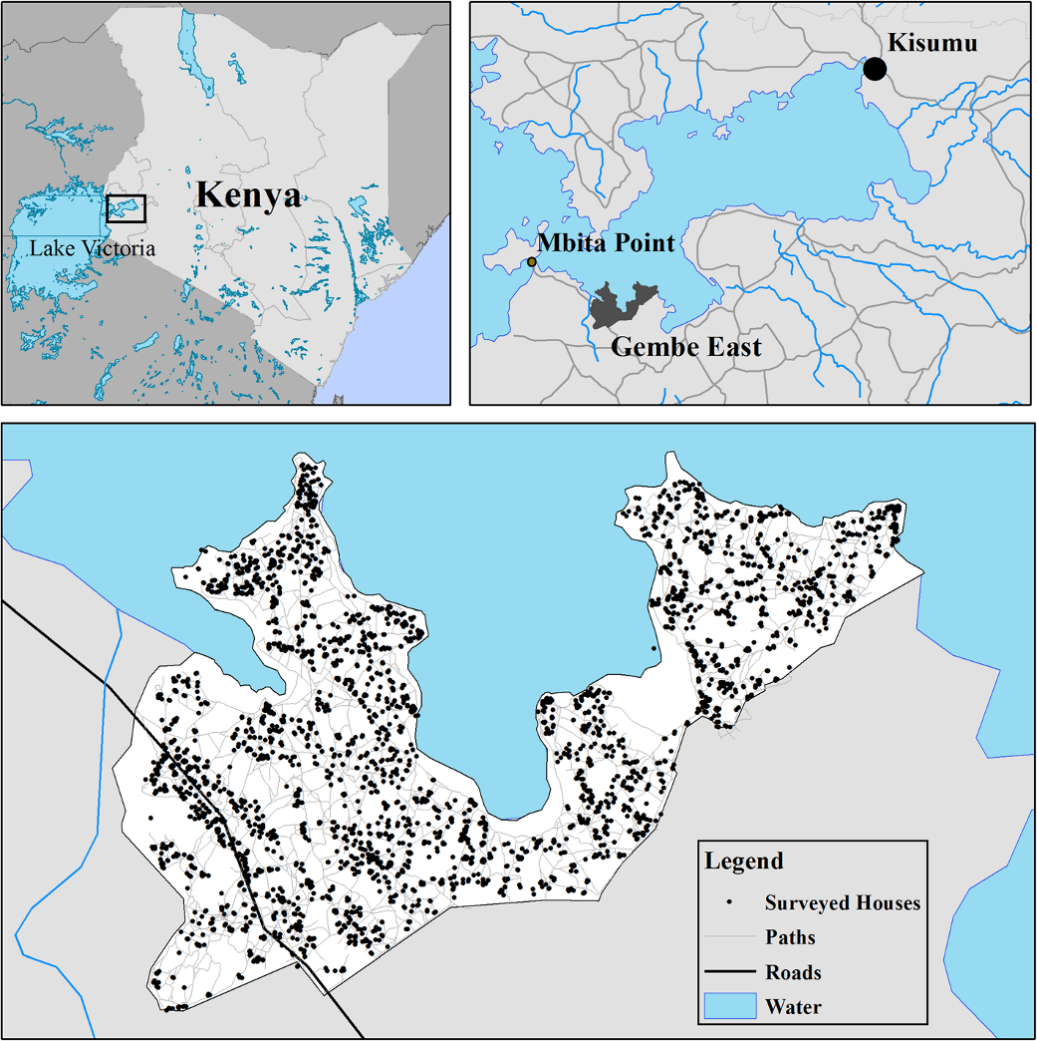
**Location of study site in the Gembe East area of Mbita District of Nyanza Province, and locations of individual households that were part of the intervention and surveillance.**

The Gembe East area of Mbita District in Nyanza Province (Figure 1) was targeted for a randomized trial of three different types of long-lasting insecticidal nets (LLINs) (Olyset® Net, Olyset® Plus, and ceiling net; Sumitomo Chemical Co. Ltd, Tokyo, Japan) in Jan/Feb 2011 [21, 22]. Teams of community members were employed to visit all households in the distribution area (N = ~4,000), collect old nets (with homeowner consent), and provide each household with new ITNs. Survey teams explained the goals and purpose of the study to adult household representatives and obtained consent. Each household received a number of marked Olyset® LLINs in relation to the number of people reported to regularly sleep in the dwelling, following WHO standards. At the time of distribution, the ages and genders of all people living in the house were noted. For each individual person, survey teams recorded whether they reported sleeping under a net the previous night. Surveyors also noted whether the person slept in a bed room or an open living room and if the person slept on a bed or the floor. This study was approved by the Ethics Committees of the Kenya Medical Research Institute (SSC No. 2131), Nagasaki University (No.10121655-2) and The University of Michigan (HUM00061464).

Between January 2012 and August 2012, field workers revisited all households in the area with the aim of assessing the condition of the nets that were distributed roughly one year prior. Permission was again requested from an available adult household representative and the survey was conducted for those households agreeing to participate. For the post-distribution round of data collection, a comprehensive, standardized, oral survey was created which included demographic questions, educational level achieved, and the occupations of male and female household heads. The survey teams recorded the presence of material goods such as radios, electricity and various types of livestock, and also noted types of roof and wall construction. From this, a composite household material wealth index was created using a principal components analysis (PCA) methodology common to analyses of socioeconomic status or households in developing countries. Membership in community groups such as churches and school attendance of children was recorded. Questions about malaria and ITN knowledge, along with general questions on health-seeking and malaria-related health behaviours were asked.

Survey workers made a complete accounting of all nets in the household. Type and condition of each net, and the age and gender of the person(s) sleeping under each, was recorded. Nets that had been previously given out in the mass distribution were identifiable through a known code written on the brand tag of the ITN, but other nets were also recorded. Field workers also documented whether or not dwellings had closed or open eaves, presence of a ceiling net, and any past administration of IRS. All data were recorded on paper and entered later into Excel. GPS coordinates for each household were recorded using a Garmin GPS 60 device.

### Statistical analyses

Spatial patterns of ITN use before and after distribution, as well as other factors, initially were assessed visually using maps produced from ArcGIS (version 10.1). GIS layers of environmental and geographic features were obtained from DIVA-GIS [23]. Spatial autocorrelation was assessed using Moran’s I statistics. All statistical analyses were performed in R (version 2.15.2).

Patterns of association of age and ITN use were graphically assessed using a local regression smoothing methodology. Bivariate associations were tested and odds ratios and confidence intervals produced using logistic regression. After testing individual bivariate associations, a backwards selection procedure was used to create an optimal multivariate model.

## Results

At the time of mass ITN distribution, 3,348 households representing 12,098 people were given nets and enrolled in the study. One year later, surveyors returned to the area and interviews were conducted with representatives from 3,493 households comprising 12,404 people. Households were located in all areas of Gembe East (Figure 1). The age of participants ranged from <1 to 99 years, with a median of 13 at the time of ITN distribution, and 15 years during the follow-up. In both phases, ~48% of people surveyed were male (Table 1).

**Table 1:** **Survey characteristics of households and people during pre-distribution (2011) and post-distribution (2012) of ITNs in Mbita District, Kenya**

Characteristic	Pre	Post	p-value
**Number of individuals**	12,098	12,404	--
**Number of households**	3,352	3,493	--
**Percent male**	48%	49%	0.29
**Median age of household members**	13	15	<0.0001
**Median number of people per household**	3	3	0.08
**Median number of nets per households**	1	2	<0.0001
**Mean number of nets per person in HH**	0.24	0.52	<0.0001
**Total percentage of people who slept under ITN the previous night**	43%	92%	<0.0001
**Mean percentage of people within households who slept under ITN the previous night**	43%	91%	<0.0001

Individual ITN use more than doubled from 43% prior to distribution to 92% one year later. Within households, the mean percentage of household members reported to have slept under an ITN the previous night increased from 40% to 75%. The median number of nets per household increased from one to two, one year post-distribution. Likewise, the number of nets per person present within each household also doubled (Table 1).

**Figure 2:**
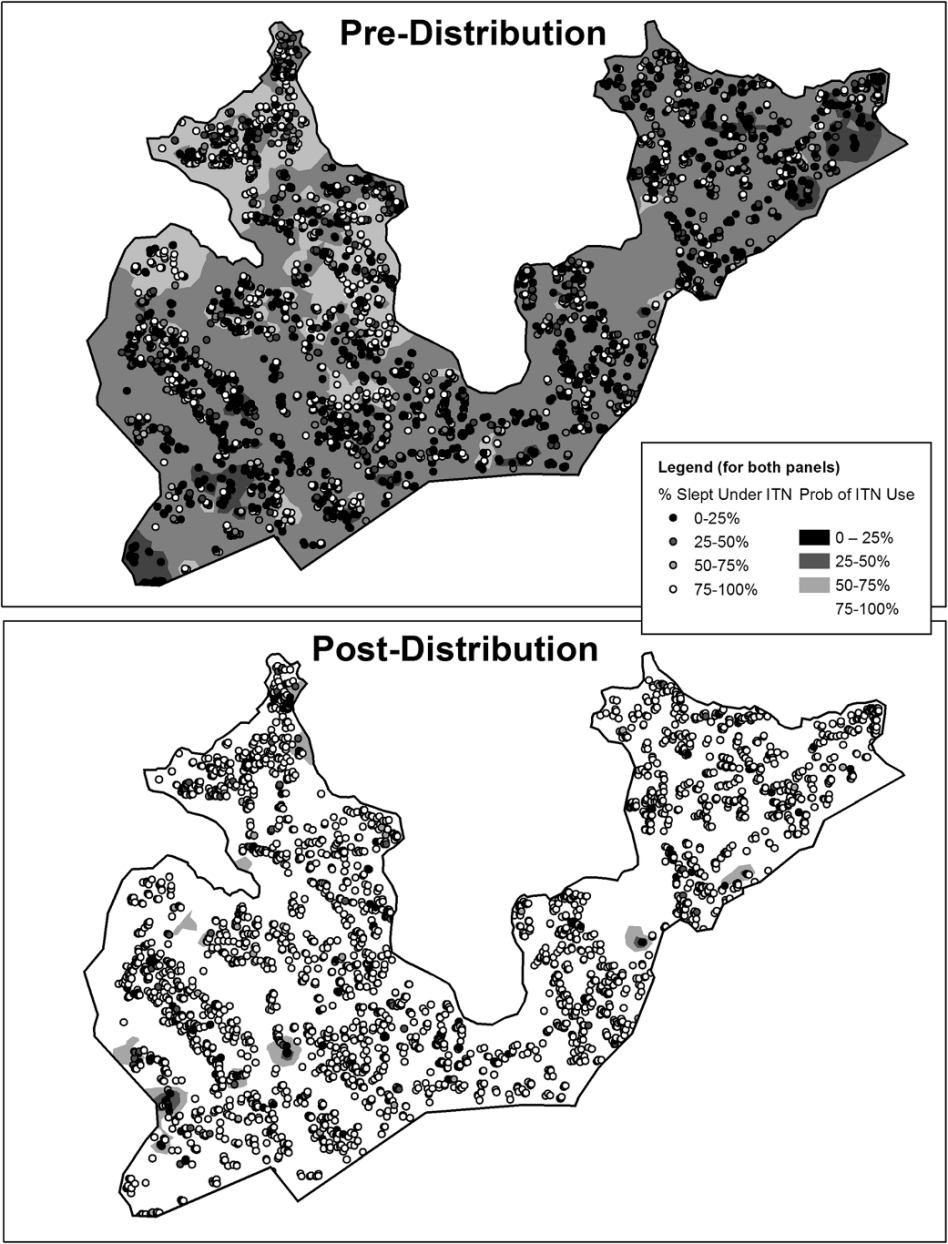
**Fraction of household members who slept under an ITN the previous evening before and after mass distribution.** Spatial patterns of ITN use are illustrated using inverse distance weighting interpolation.

**Figure 3:**
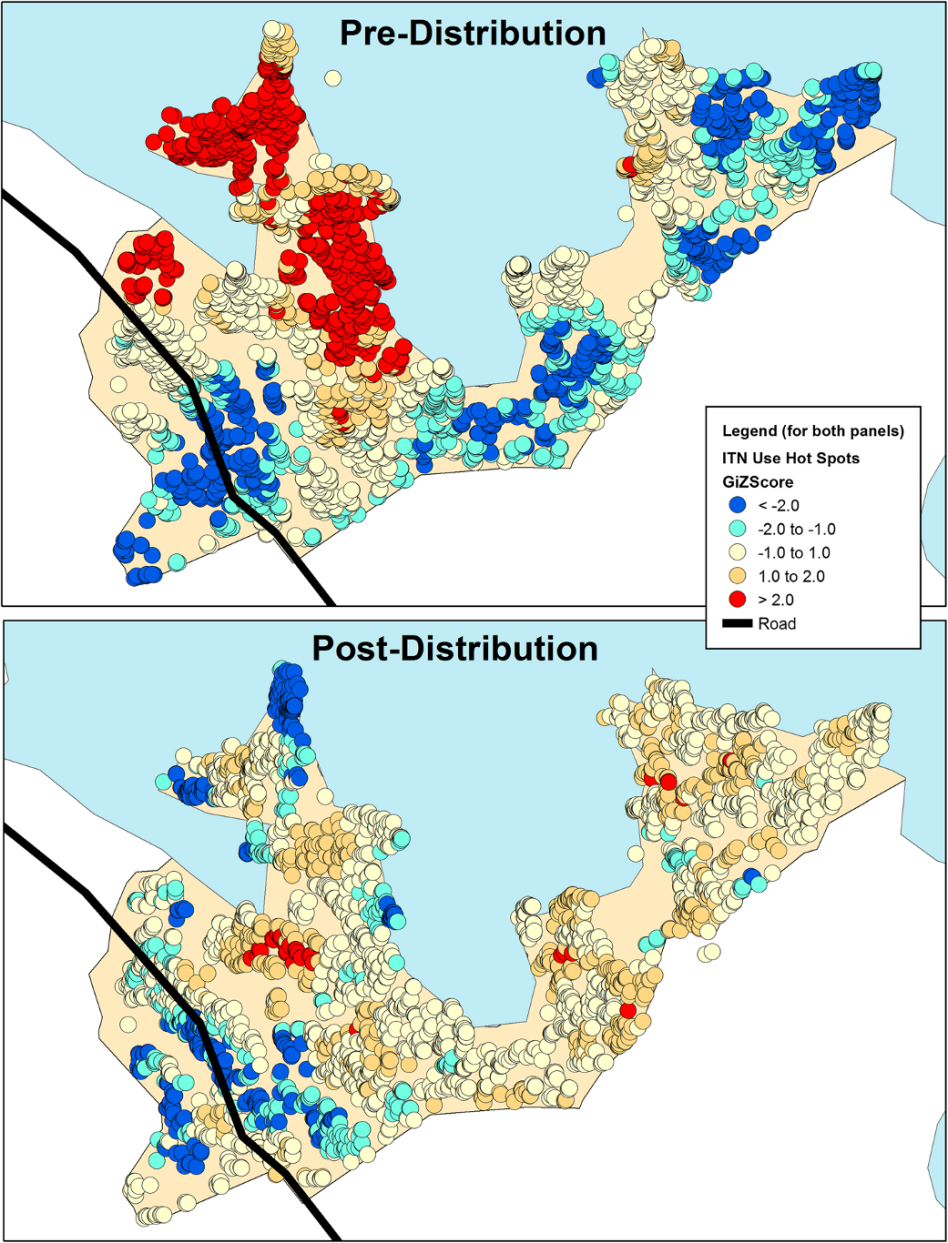
Getis-Ord Gi* statistics for each household to measure spatial clustering of household ITN use as a percentage of total number of residents. Colors represent areas of statistically significantly high levels of ITN use (red) and significantly low levels of ITN use (blue).

The spatial distribution of the percentage of household members reporting ITN use the previous night differed between the two periods (Figure 2). Before distribution, use was low, though apparently slightly elevated in areas closer to water. One year following distribution, overall use increased, with some areas showing close to 100% compliance among household members. Statistical tests confirmed the presence of household-level clusters (spatial autocorrelation) of net use prior to the ITN distribution campaign. One year after ITNs were provided, spatial clustering was still evident, though to a lesser degree (Moran’s I statistic: Pre: .158, p < 0.0001, Post: .027, p = 0.0001). Knowing this, ArcGIS was used to calculate the Getis-Ord Gi* statistic, a measure of spatial clustering of high and low values within a dataset. The Gi* stat was plotted for each household, both before and after the mass distribution (see Figure 3). Prior to distribution high levels of ITN use were found in the areas close to the water on the western side of the area. Low levels of use were found inland nearby a paved road and in other parts of the area. Following distribution, clusters of extraordinarily high coverage were few, likely because most the residents of most households reported sleeping under an ITN. However, clusters of households where a significantly lower percentage of persons slept under an ITN were to be found again by the paved road and also small areas close to the water on the western side.

### Condition and number of ITNs following distribution

**Figure 4:**
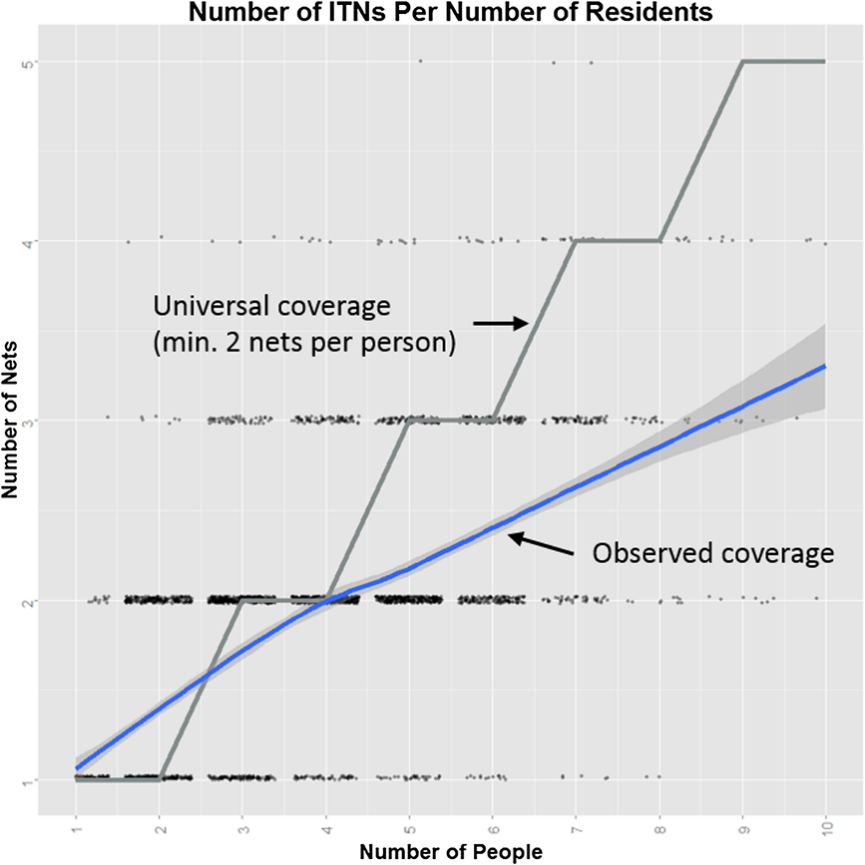
**Number of ITNs by the number of people living in the home.** Gray line represents the expected trend of ownership given the number of people and a criterion for universal coverage which assumes at least one net per two people. Blue line (with confidence bands) indicates the observed trend.

Nearly all (97%) of the nets present at households during the second survey were marked Olyset® nets that had been distributed one year earlier, with 99% of these being found in current use inside the dwelling (as opposed to outdoors). About 20% of nets, the vast majority of which were in new condition one year previously, had visible holes (Table 2). Survey teams attempted to assess presence of stored/unused nets and possible diversion of nets for other purposes, but this proved difficult as many diverted nets were present in areas other than the home. ITNs were initially distributed in a manner that would allow for universal coverage, with a goal of providing at least one net per two people in the household. Households with only one or two members were given a single net, three- or four-member households were given two, etc. However, upon follow up, it was found that only 59% of households possessed sufficient nets to satisfy the universal coverage criterion. A graphic comparison of the actual number of nets per resident to the predicted ratio of nets under the universal coverage criterion can be found in Figure 4.

**Table 2:** **Characteristics of ITNs in households sampled during post-distribution (2012) survey in Mbita District, Kenya**

Characteristic		N	Percent
**Total No. of Nets**		5,733	100%
**Type of Net**	**Olyset**	5,534	97%
	**Permanet**	142	3%
	**Other**	12	0%
**How Obtained**	**Nagasaki**	4,772	89%
	**Bought**	207	4%
	**Other**	404	8%
**In the House**	**Yes**	5,432	99%
	**No**	76	1%
**Currently In Use**	**Yes**	5,235	96%
	**No**	215	4%
**Holes**	**Yes**	1,051	19%
	**No**	4,369	81%

### Determinants of ITN use

**Figure 5:**
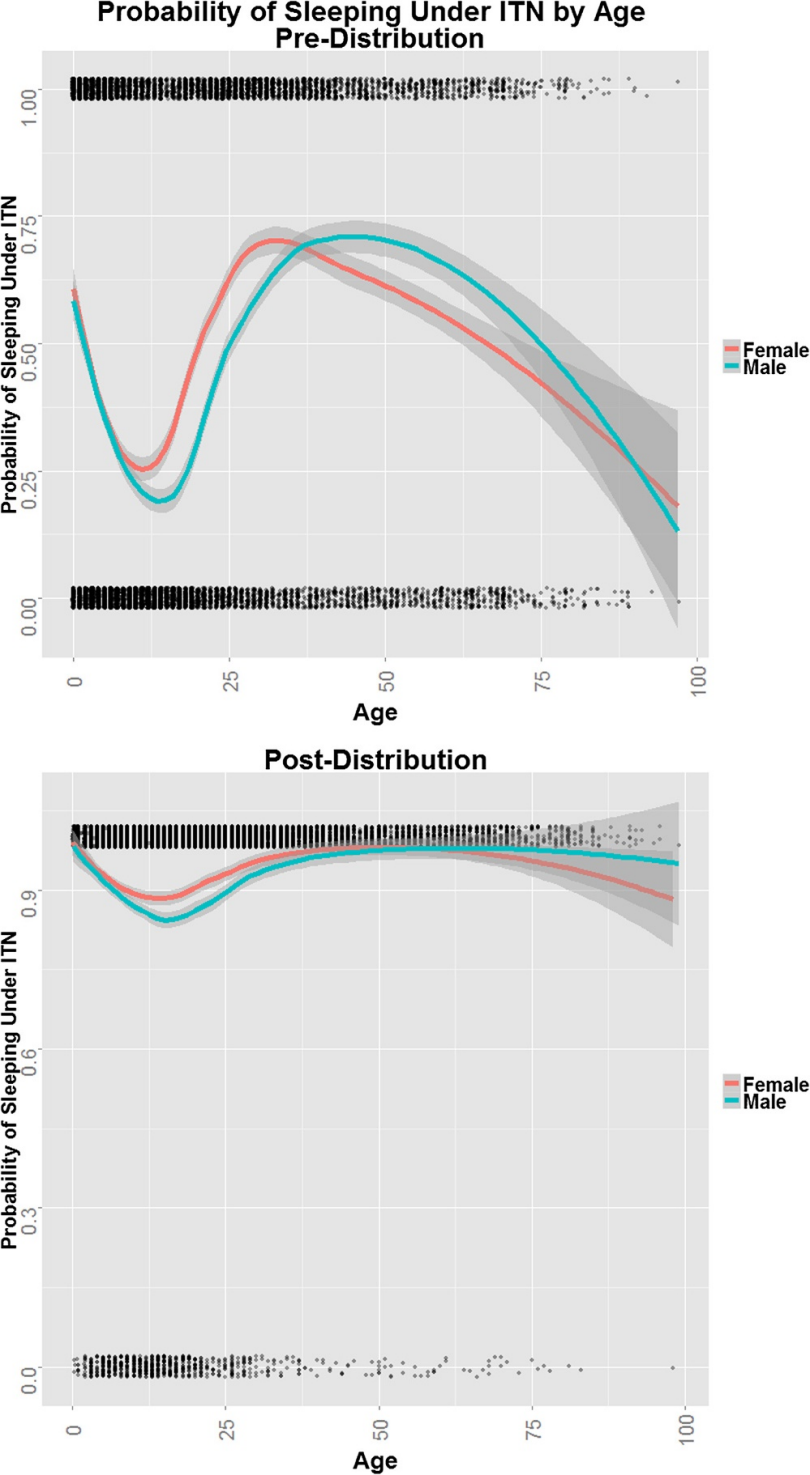
**Patterns of ITN use by age both before and after mass distribution.** Lines are produced using a local regression smoothing technique. Patterns of males and females are presented for comparison.

At the time of distribution, ITN use overall was reported by less than half of surveyed people, but heterogeneous among age groups (Figure 5). Reported age-specific use of an ITN was generally high just after birth, declined to very low levels in the late teen years, rose again until approximately age 30, and then dropped again among the elderly. Increasing use among males in their 20s occurred later than that among females, perhaps reflecting the tendency for men to be older than women in couples of reproductive age. Although coverage of ITNs one year post-distribution was nearly universal, the age-specific pattern of ITN use, overall and by gender, was the same as that observed before net distribution. Females generally tended to use ITNs more than males (F: 93% *vs* M: 90%, p < 0.0001), possibly the result of previous ITN distributions and health education campaigns which targeted mothers and pregnant women.

### Household survey

Overall, 57.6% of individuals reported sleeping on the floor as opposed to a bed, and 39.4% reported sleeping in open rooms rather than a formal bedroom (Table 3). Nearly 90% of all houses surveyed were observed to have open eaves, which would increase mosquito access to humans at night. Ceiling nets, which are intended to cover open eaves and prevent mosquito entry, were distributed to certain target areas as part of an unrelated randomized trial. ~35% of households surveyed had ceiling nets. The Kenyan Ministry of Health had also performed indoor residual spraying within the year that nets were distributed. ~78% of households had been sprayed. Surveyors observed that ~5% of households were using nets for other purposes, such as fishing, covering plants and as garden fences. Fathers tended to be have higher levels of formal schooling than mothers, and occupational activities differed slightly (Table 3).

**Table 3:** **Results of the household survey**

Characteristic	Classification	No.	Percent
**Eaves**	Covered	369	11%
	Open	2,908	89%
**Ceiling net present**	Yes	705	34%
	No	1,342	66%
**Home was sprayed in the last year (IRS)**	Yes	2,060	78%
	No	587	22%
**Use of ITNs for purposes other than malaria prevention**	Yes	137	5%
	No	2,612	95%
**Responded that the purpose of an ITN is to prevent malaria**	Yes	481	17%
	No	2,324	83%
**Responded that the purpose of an ITN is to prevent mosquitoes from biting**	Yes	2,421	86%
	No	384	14%
**Husband education**	Never	90	5%
	Primary	1,299	68%
	Secondary	326	17%
	College	197	10%
**Husband occupation**	Fishing	850	43%
	Farmer	361	18%
	Merchant	294	15%
	Other	208	10%
	Teacher	182	9%
	None	104	5%
	Government	1	0%
**Wife education**	Never	393	15%
	Primary	1,866	73%
	Secondary	217	9%
	College	70	3%
**Wife occupation**	Merchant	1,034	43%
	None	877	36%
	Farmer	319	13%
	Other	96	4%
	Teacher	62	3%
	Fishing	17	1%

### Determinants of ITN use pre- and post-distribution

Numbers of people who lived in households with particular characteristics and percent of people who used ITNs are presented in Additional file 1. Bivariate logistic regression models for the pre- and post-distribution (Additional file 2) were constructed to allow for significance testing. Prior to distribution, males were less likely to sleep under an ITN than females [OR 0.47 (0.33, 0.67)]. The odds of using an ITN increased for every extra person residing in the home [OR 1.36 (1.16, 1.60)]. There were heterogeneities in ITN use among age groups, with 5 to 18 year olds having the lowest odds of using an ITN, compared with under 5′s [OR 0.36 (0.28, 0.48)].

Sleeping on the floor was associated with a decreased odds of using an ITN [OR 0.002 (0.001, 0.003)] along with sleeping in an open room [OR 0.002 (0.002, 0.003)] and having received IRS in the past [OR: 1.97 (1.25, 3.09)]. Individuals in households which were observed to use ITNs for purposes other than malaria control were more likely to sleep under an ITN [OR .2.69 (1.29, 5.63)]. Paternal education was not associated with ITN use pre-distribution while maternal education was. There was suggestive evidence that young people in households where the father was involved with fishing were more likely to use ITNs than households where the father was involved with other occupations. Maternal occupation was not associated with ITN use pre-distribution. Graded and inverse associations of material wealth with ITN use prior to the distribution programme were found.

Following ITN distribution, males were still less likely to sleep under ITNs than females [OR 0.61, (0.53, 0.70)]. As before, sleeping on the floor and sleeping in an open room were both highly associated with decreased odds of ITN use. It is also noted the similar age effects between the two distribution periods. 5 to 18 year olds were still the group least likely to use ITNs, compared with under 5′s [OR 0.25 (0.14, 0.41)]. Open eaves [OR 0.79 (0.00, 137.44)], an incremental increase in the number of rooms in the household, alternative uses of ITNs [OR 0.45 (0.00, 4082.40)], and indoor residual spraying [OR 2.58 (0.07, 93.32)] were no longer associated with ITN use. Maternal and paternal education and occupation were not associated with ITN use post-distribution. The positive association of material wealth with ITN use prior to the intervention disappeared following the mass distribution of ITNs. See Additional file 2 for complete results.

It is noted that in both the pre- and post-distribution phases, the odds ratios for sleeping location and room choice are very small. This is the result of including a random effect for household. Pre-distribution, sleeping on the floor [OR 0.24 (0.22, 0.26)] and sleeping in an open room [OR 0.22 (0.20, 0.25)] were associated with decreased odds of sleeping under and ITN. Likewise, post-distribution, sleeping on the floor [OR 0.31 (0.26, 0.36)] and sleeping in an open room [OR 0.30 (0.26, 0.35)] were associated with decreased odds of using ITNs. It should be noted that more than 91% of people who slept in an open room slept on the floor in both phases. When accounting for household effects of ITN use, which are a result of limited space and numbers of ITNs, sleeping on the floor was almost wholly associated with not using and ITN. Similarly, the large odds ratio was tempered somewhat when no including the random effect for household, but the age group of 30+ was still the group most likely to use ITNs compared to small children (pre [OR 1.83 (1.61, 2.08)], post [OR 1.69 (1.28, 2.24)]). When accounting for overall net use in the household, the effects of age are accentuated.

## Discussion

These results demonstrate that high levels of ITN coverage can be obtained through mass distribution of free nets, and that use compliance can be rapidly increased and maintained at least one year post-distribution. Inequities in coverage among socio-economic, educational and occupational groups can be erased through comprehensive no-payment provision of ITNs. Furthermore, problems of spatial heterogeneities in ITN possession can be successfully mitigated through a direct-to-household delivery strategy.

However, evidence suggests that even when sufficient numbers of nets are proactively provided to cover all residents in each household, patterns of failing to use them may persist. In this study, despite dramatically increased levels of ITN compliance overall, age-specific differences in ITN use remained unchanged. Heterogeneous age effects in ITN use have been noted by other researchers in a variety of contexts, including Kenya [5]. However, it has been thought that distribution strategies which target children and pregnant women are responsible for such patterns of underuse. This study, where nets were provided to all members of the community, provides evidence to counter that assumption. In addition, practical issues of household sleeping arrangements and home construction continued to present barriers to full ITN compliance. Constraints on where and how to hang nets in common areas, a hurdle noted in other studies [24–26], are not, however, easily rectified.

Another concern that was discovered is the problem of ITN “leakage”, an issue that has been noted in other studies [27–29]. Although sufficient numbers of ITNs were given to cover all household members, the actual number of nets found one year later was much lower than what was initially provided. During related research, we found that freely distributed ITNs can be found in households other than those to which they were initially given (unpublished observation). Furthermore, even though nets are provided with the message that are specifically to be used for malaria prevention, household economic needs lead to nets being diverted to remunerable activities such as fishing or agriculture [30]. It is unreasonable to expect that household heads, weighing numerous and sometimes conflicting pressures, will follow the requests of malaria researchers or public health workers. Thus, the possibility of leakage should be considered prior to mass distribution campaigns. Despite some net use for non-malaria prevention purposes, more than 90% of people slept under an ITN, and more than 97% of the nets found in the households were from this distribution campaign. Programmatically, this should be considered to be a success.

A “hot spot” analysis of ITN use was performed both before and after the distribution. It was found that before the distribution, high household level coverage of ITNs was found in the wet areas near the lake, possibly reflecting prior targeted intervention efforts. Further research, though, might also explore how mosquito density impacts ITN use. Household members in this area might tend to use nets more than in other areas simply due to an unbearable number of biting mosquitoes. Both before and after the intervention, ITN use in households along the paved highway was significantly lower than in other areas. Though it was not explored in this study, three possible explanations could be considered. First, households along the paved road often operate as storefronts so that sleeping spaces within the structure might be inadequate for hanging nets. It could also be the case that the storefronts only act as seasonal or temporary dwellings. Household members maybe have residences in other locations.

Maintaining consistent coverage of ITNs within transient households might present a challenge to malaria control programmes and should be considered for future research efforts. Second, it is possible that houses along the paved road are of sufficient quality to prevent mosquito entry through the presence of window screens, an important piece of data which was not collected. Third, the developed nature of this area might negatively impact breeding sites so that mosquito densities are low. More work should be done to assess the precise factors, which determine net use (or non-use) in this type of area.

Although net use was reported by a household member who may have under-reported use to obtain a free net, the age patterns that we observed agreed with other similar studies [5, 31, 32]. If actual use was understated this would have merely shifted the curve downward, but not differentially. That reported use following free distribution was nearly universal, though, is inconsistent with underreporting. In addition, surveillance teams visually confirmed whether nets were hanging, and different types of questions explored who was sleeping under nets. Thus, it is probable that this research accurately captured the general patterns of net presence and use.

Data from only two time points limits the ability to consider net use patterns in different seasons or over multiple years. Although data were collected over several months, this occurred at about the same time of year before and after net distribution. The tests for spatial patterns of ITN use, however, did not reveal patterns suggesting that sampling was time-dependent. Continued monitoring of net use should provide further insights into the duration of campaign impacts.

The extent of increased ITN coverage and use in this study provides reason for hope that such programmes will reduce malaria incidence. It is expected that *Plasmodium* transmission should decline, incidence of disease be lessened, and malaria-related mortality averted. However, given the very intense transmission of this area [33, 34], the persistence of diminished ITN use by specific age groups is cause for great concern. Like all African countries, Kenya’s age distribution is bottom heavy. If large numbers of young people are not using nets, even in the context of widespread availability, this subpopulation could contribute substantially to sustained transmission compromising control efforts.

## Electronic supplementary material

Additional file 1: **ITN use in individuals given household level and individual variables pre and post distribution.** Absolute numbers and percentages are provided. (DOCX 18 KB)

Additional file 2: **Results of bivariate models of ITN use including random effect for household for pre and post ITN distribution.** Odds ratios are presented with 95% confidence intervals. (DOCX 20 KB)
