# Supplementary material for: Insecticide-treated net use before and after mass distribution in a fishing community along Lake Victoria, Kenya: successes and unavoidable pitfalls
Source: Malar J. 2014 Nov 28;13:466. doi: 10.1186/1475-2875-13-466 (PMC4289357; doi:10.1186/1475-2875-13-466)
Supplement: Supplementary file 1 — Additional file 1: ITN use in individuals given household level and individual variables pre and post distribution. Absolute numbers and percentages are provided. (DOCX 18 KB) [file 12936_2014_3656_MOESM1_ESM.docx]

Additional file 1: ITN use in individuals given household level and individual variables pre and post distribution. Absolute numbers and percentages are provided.

|  | **Pre-Distribution** | | **Post-Distribution** | |
| --- | --- | --- | --- | --- |
|  | **No** | **Yes** | **No** | **Yes** |
| **Gender** |  |  |  |  |
| **F** | 2645(55%) | 2199(45%) | 371(7%) | 5069(93%) |
| **M** | 2638(60%) | 1780(40%) | 471(9%) | 4692(91%) |
| **Sleeping Arrangement** |  |  |  |  |
| **Bed** | 2072(42%) | 2869(58%) | 245(4%) | 5640(96%) |
| **Floor** | 3135(75%) | 1057(25%) | 544(13%) | 3758(87%) |
| **Sleeping Room** |  |  |  |  |
| **Bed room** | 2204(42%) | 3033(58%) | 273(4%) | 6010(96%) |
| **Open room** | 3030(76%) | 932(24%) | 523(13%) | 3528(87%) |
| **Eaves** |  |  |  |  |
| **Covered** | 518(50%) | 522(50%) | 71(6%) | 1058(94%) |
| **Open** | 4576(58%) | 3304(42%) | 693(8%) | 8304(92%) |
| **Age** |  |  |  |  |
| **0-5** | 1059(58%) | 753(42%) | 118(6%) | 1863(94%) |
| **5-18** | 2339(74%) | 806(26%) | 450(12%) | 3374(88%) |
| **18-30** | 613(41%) | 888(59%) | 159(8%) | 1866(92%) |
| **30+** | 816(43%) | 1063(57%) | 92(4%) | 2489(96%) |
| **Ceiling Net** |  |  |  |  |
| **No** | 2119(59%) | 1454(41%) | 361(9%) | 3777(91%) |
| **Yes** | 1379(59%) | 950(41%) | 109(5%) | 2123(95%) |
| **Indoor Residual Spraying (IRS)** |  |  |  |  |
| **No** | 833(61%) | 535(39%) | 190(12%) | 1376(88%) |
| **Yes** | 3257(56%) | 2526(44%) | 423(6%) | 6302(94%) |
| **Alternative Use of ITNs** |  |  |  |  |
| **No** | 3975(55%) | 3192(45%) | 632(8%) | 7787(92%) |
| **Yes** | 284(61%) | 178(39%) | 22(5%) | 451(95%) |
| **Husband Education** |  |  |  |  |
| **Never** | 137(53%) | 122(47%) | 18(8%) | 213(92%) |
| **Primary** | 2000(50%) | 1963(50%) | 262(6%) | 4465(94%) |
| **Secondary** | 484(55%) | 404(45%) | 108(10%) | 1007(90%) |
| **College** | 321(57%) | 247(43%) | 35(5%) | 657(95%) |
| **Wife Education** |  |  |  |  |
| **Never** | 621(65%) | 340(35%) | 102(11%) | 857(89%) |
| **Primary** | 2996(54%) | 2603(46%) | 438(7%) | 6150(93%) |
| **Secondary** | 279(50%) | 282(50%) | 57(8%) | 661(92%) |
| **College** | 89(62%) | 54(38%) | 18(8%) | 198(92%) |
| **Husband Occupation** |  |  |  |  |
| **Farmer** | 626(54%) | 533(46%) | 75(6%) | 1138(94%) |
| **Fishing** | 1171(48%) | 1287(52%) | 196(6%) | 2972(94%) |
| **Merchant** | 428(52%) | 401(48%) | 63(7%) | 878(93%) |
| **None** | 158(61%) | 100(39%) | 16(6%) | 247(94%) |
| **Other** | 337(60%) | 226(40%) | 74(11%) | 619(89%) |
| **Teacher** | 298(57%) | 227(43%) | 31(5%) | 618(95%) |
| **Wife Occupation** |  |  |  |  |
| **Farmer** | 600(58%) | 443(42%) | 58(6%) | 985(94%) |
| **Fishing** | 15(47%) | 17(53%) | 3(5%) | 63(95%) |
| **Merchant** | 1782(56%) | 1385(44%) | 229(6%) | 3466(94%) |
| **None** | 1243(53%) | 1088(47%) | 198(7%) | 2592(93%) |
| **Other** | 152(50%) | 152(50%) | 38(12%) | 283(88%) |
| **Teacher** | 70(57%) | 52(43%) | 17(8%) | 185(92%) |
| **SES Quintile** |  |  |  |  |
| **5 (Least Poor)** | 1260(53%) | 1106(47%) | 146(6%) | 2363(94%) |
| **4** | 1166(57%) | 875(43%) | 168(7%) | 2099(93%) |
| **3** | 1073(58%) | 789(42%) | 171(8%) | 2005(92%) |
| **2** | 956(60%) | 632(40%) | 147(8%) | 1736(92%) |
| **1 (Poorest)** | 873(59%) | 607(41%) | 218(12%) | 1664(88%) |
