# Supplementary material for: Insecticide-treated net use before and after mass distribution in a fishing community along Lake Victoria, Kenya: successes and unavoidable pitfalls
Source: Malar J. 2014 Nov 28;13:466. doi: 10.1186/1475-2875-13-466 (PMC4289357; doi:10.1186/1475-2875-13-466)
Supplement: Supplementary file 2 — Additional file 2: Results of bivariate models of ITN use including random effect for household for pre and post ITN distribution. Odds ratios are presented with 95% confidence intervals. (DOCX 20 KB) [file 12936_2014_3656_MOESM2_ESM.docx]

Additional file 2

|  | **Pre-Distribution** | |  | **Post-Distribution** | |
| --- | --- | --- | --- | --- | --- |
| **Characteristics** | **Odds Ratio (CI)** | **p-value** |  | **Odds Ratio (CI)** | **p-value** |
|  |  |  |  |  |  |
| **Male (vs. Female)** | 0.47 (0.33, 0.67) | **<0.0001** |  | 0.61 (0.53, 0.70) | **<0.0001** |
| **Number of People (in 1’s)** | 1.36 (1.16, 1.60) | **<0.0001** |  | 1.43 (0.66, 3.10) | 0.36 |
| **Slept on Floor (vs. Bed)** | 0.002 (0.001, 0.003) | **<0.0001** |  | 0.0000 (0.0000, 0.0005) | **<0.0001** |
| **Slept in Open Room (vs. Bed Room)** | 0.002 (0.002, 0.003) | **<0.0001** |  | 0.0001 (0.0000, 0.0006) | **<0.0001** |
| **Open Eaves** | 0.41 (0.25, 0.67) | **<0.0001** |  | 0.79 (0.00, 137.44) | 0.93 |
| **Number of Rooms (in 1’s)** | 1.29 (1.09, 1.52) | **0.002** |  | 1.38 (0.31, 6.23) | 0.68 |
| **Age** |  | **<.0001** |  |  | **<.0001** |
| **0-5** | ref |  |  | ref |  |
| **5-18** | 0.36, (0.28, 0.48) | **<.0001** |  | 0.25, (0.14, 0.41) | **<.0001** |
| **18-30** | 11.31, (8.18, 15.65) | **<.0001** |  | 2.90, (1.39, 6.05) | 0.004 |
| **30+** | 19.73, (13.82, 28.16) | **<.0001** |  | 200.48, (57.55, 698.43) | **<.0001** |
| **Malaria Protection** |  |  |  |  |  |
| **Ceiling Net** | 1.22 (0.80, 1.88) | 0.35 |  | 2.06 (0.02, 192.19) | 0.76 |
| **IRS** | 1.97 (1.25, 3.09) | **0.003** |  | 2.58 (0.07, 93.32) | 0.61 |
| **Alternative Use of ITNs** | 2.69 (1.29, 5.63) | **0.01** |  | 2.22 (.17, 27.82) | 0.86 |
| **Husband Education** |  | <.028 |  |  | .99 |
| **Never** | ref |  |  | ref |  |
| **Primary** | 1.42 (0.56, 3.62) | 0.46 |  | 2.14 (0.00, 7352.73) | 0.85 |
| **Secondary** | 0.69 (0.24, 1.95) | 0.48 |  | 1.10 (0.00, 6033.05) | 0.98 |
| **College** | 0.70 (0.23, 2.12) | 0.53 |  | 1.92 (0.00, >10000.00) | 0.9 |
| **Wife Education** |  | **.001** |  |  | .98 |
| **Never** | ref |  |  | ref |  |
| **Primary** | 2.61 (1.62, 4.22) | **<.0001** |  | 2.14 (0.05, 83.92) | 0.68 |
| **Secondary** | 2.78 (1.33, 5.84) | **0.01** |  | 1.48 (0.01, 395.33) | 0.89 |
| **College** | 1.36 (0.40, 4.59) | 0.62 |  | 1.65 (0.00, >10000.00) | 0.92 |
| **Husband Occupation** |  | **<.0001** |  |  | .99 |
| **Farming** | ref |  |  | ref |  |
| **Fishing** | 1.78 (1.02, 3.10) | **0.04** |  | 1.01 (0.01, 203.58) | 1 |
| **Merchant** | 1.01 (0.50, 2.02) | 0.98 |  | 0.93 (0.00, 939.45) | 0.98 |
| **Teacher** | 0.66 (0.29, 1.50) | 0.32 |  | 1.39 (0.00, 7513.40) | 0.94 |
| **None** | 0.31 (0.11, 0.85) | **0.02** |  | 0.85 (0.00, >10000.00) | 0.97 |
| **Other** | 0.33 (0.15, 0.73) | **0.01** |  | 0.55 (0.00, 413.04) | 0.86 |
| **Wife Occupation** |  | .56 |  |  | .99 |
| **Farming** | ref |  |  | ref |  |
| **Fishing** | 2.51 (0.17, 36.32) | 0.5 |  | 0.80 (0.00, >10000.00) | 0.98 |
| **Merchant** | 1.24 (0.73, 2.11) | 0.42 |  | 1.00 (0.01, 127.34) | 1 |
| **Teacher** | 1.50 (0.40, 5.56) | 0.54 |  | 0.77 (0.00, >10000.00) | 0.96 |
| **None** | 1.65 (0.96, 2.83) | 0.07 |  | 0.84 (0.01, 116.49) | 0.94 |
| **Other** | 2.03 (0.78, 5.26) | 0.14 |  | 0.53 (0.00, 1052.27) | 0.87 |
| **Wealth Quintiles** |  | .0001 |  |  | .99 |
| **5 (Least Poor)** | ref |  |  | ref |  |
| **4** | 0.57 (0.36, 0.90) | **0.02** |  | 1.09 (0.01, 134.57) | 0.97 |
| **3** | 0.52 (0.33, 0.84) | **0.007** |  | 1.06 (0.01, 117.78) | 0.98 |
| **2** | 0.35 (0.21, 0.57) | **<0.0001** |  | 0.83 (0.01, 88.67) | 0.94 |
| **1 (Poorest)** | 0.32 (0.19, 0.52) | **<0.0001** |  | 0.50 (0.01, 40.96) | 0.76 |
